# Subtype matters: ovarian endometriosis impairs ovarian reserve and embryo quality—should these patients consider fertility preservation?

**DOI:** 10.1007/s00404-026-08446-8

**Published:** 2026-06-10

**Authors:** C. Meyer, C. Staib, S. Löb, A. Altides, M. Schwab, J. Büchel, A. Scherer-Quenzer, M. Kiesel, A. Wöckel, S. L. Herbert

**Affiliations:** Department of Gynecology and Obstetrics, University Hospital Würzburg, Josef-Schneider-Straße 4, 97080 Würzburg, Germany

**Keywords:** Endometriosis, AMH, Diminished ovarian reserve, Medical freezing, Personalized medicine

## Abstract

**Research question:**

Which patients with endometriosis suffer from diminished ovarian reserve as well as impaired embryo quality and therefore could benefit from medical freezing as part of fertility preservation strategies?

**Design:**

This retrospective study analyzed 205 patients who underwent follicle puncture at our center in preparation for IVF/ICSI treatment. All patients with laparoscopically confirmed endometriosis were classified according to rASRM and #ENZIAN. In total, 183 follicle punctures and 168 embryos were evaluated. Anti-Müllerian hormone (AMH) levels, the antral follicle count (AFC), the number of retrieved oocytes as well as the rate of mature oocytes and the fertilization rate were compared among the different subtypes of endometriosis. Embryo quality was assessed by the KIDScore™ on days 3 and 5.

**Results:**

The analyses revealed significant differences in the AFC among patients with peritoneal (mean AFC: 16.43), deep-infiltrating (11.84) and ovarian endometriosis (10.85) (*p* = 0.006). The largest difference was observed between superficial and ovarian endometriosis (*p* = 0.004). The number of retrieved oocytes also differed significantly among the subgroups (*p* = 0.012), with the strongest contrast between deep-infiltrating (11.18) and ovarian endometriosis (8.14). Although the AFC, AMH and number of retrieved oocytes were strongly correlated, AMH alone did not differ significantly between the subgroups. The rate of mature oocytes was the lowest in patients with deep-infiltrating endometriosis but did not reach statistical significance. Patients with endometriomas presented the lowest fertilization rates and KIDScore™ values; however, only the difference in KIDScore™ D5 reached statistical significance (FR: *p* = 0.077, D3: *p* = 0.659, D5: *p* = 0.005).

**Conclusions:**

Endometriosis subtypes differ in their impact on the ovarian reserve. Patients with ovarian endometriosis exhibit a diminished ovarian reserve, reflected by a lower AFC, a lower number of retrieved oocytes and lower number of mature oocytes. Additionally, a lower embryo quality was also observed. These findings could not be replicated in patients with superficial or deep-infiltrating endometriosis. Our study highlights the importance of identifying the specific form of endometriosis. Given that diminished ovarian reserve and recued embryo quality were observed at the time of reproductive therapy, we propose that early elective oocyte cryopreservation may help prevent these adverse outcomes, particularly in patients with ovarian endometriosis. However, additional factors and fertility preservation strategies should be taken into account when considering its indication and fertility preservation strategy in patients with deep-infiltrating or superficial endometriosis.

## What does this study adds to the clinical work

**Table:** 

This study analyzed the ovarian reserve across different subtypes of endometriosis. In clinical practice, assessing the specific form of endometriosis is important for developing a personalized therapeutic concept. Medical freezing for patients with endometriomas may be indicated based on ovarian reserve markers such as AMH-levels and AFC. In contrast, in other endometriosis subtypes, especially deep-infiltrating endometriosis, additional factors must be taken into consideration.	

## Introduction

Endometriosis is one of the most common gynecological conditions, affecting approximately 10% of women of reproductive age [1]. It is defined by the presence of estrogen-dependent endometrial-like tissue outside the uterine cavity [2]. The typical symptoms of endometriosis include chronic pelvic pain, dysmenorrhea, dyspareunia, dyschezia and dysuria, as well as impaired fertility [1, 3].

Approximately 50% of infertile women are diagnosed with endometriosis [4]. While the monthly fecundity rate in healthy fertile women ranges from 15–25%, it decreases to only 2–5% in those with endometriosis [5–8]. Additionally, endometriosis is associated with an increased risk of miscarriages and a reduced live birth rate [5, 9]. The precise mechanisms by which fertility is impaired are not completely understood. A multifactorial interplay likely involves mechanical disruptions that hinder oocyte release and transportation through the fallopian tube, altered sperm motility and disordered uterine peristalsis. Also, the influence of inflammatory cytokines, angiogenic factors, immune cells, and both genetic and environmental factors are discussed as factors for the reduced fertility of patients suffering from endometriosis [8]. Severe endometriosis, characterized by extensive pelvic adhesions and an obliterated rectouterine pouch, can lead to infertility due to tubal occlusion and mechanical barriers that prevent the oocyte from being released from the ovary and hinder spermatozoa from moving to the fallopian tube [8, 10, 11]. The hemolysis of endometriomas results in iron release, which stimulates the production of cytokines and reactive oxygen species (ROS). ROS induce fibrosis of the ovarian cortex, leading to hypoperfusion and the destruction of primordial follicles. Surgical treatment of endometriomas can also lead to an iatrogenic reduction in ovarian tissue and thus contribute to a diminished ovarian reserve [12–14]. The peritoneal fluid of patients with endometriosis contains relatively high concentrations of proinflammatory substances, which impair oocyte and embryonal quality and reduce the levels of growth factors in oocytes, leading to altered folliculogenesis and decreased oocyte developmental competence, and lowering the fertilization rate [15, 16]. In addition, ROS in the peritoneal fluid might have a negative effect on embryos by initiating inflammatory responses, peroxidizing membrane lipids, and damaging both the cytoskeleton and DNA integrity [17, 18]. Women with endometriosis also exhibit a lower implantation rate, partly due to an impaired receptivity of the endometrium to estradiol and progesterone. A reduced expression of progesterone receptors has been observed in affected patients, which might lead to a disrupted transformation of the endometrium from the proliferative phase to the secretory and receptive phases [19, 20].

The management of endometriosis-related infertility typically involves three main modalities: medical treatment, surgery, and in vitro fertilization/ intracytoplasmic sperm injection (IVF/ICSI). The most appropriate approach depends on the stage of endometriosis, the subtype of endometriosis, the sperm quality and the age of the patient. The Endometriosis Fertility Index (EFI) can be used to assess these factors and predict the chance of pregnancy [10, 21]. Surgical intervention may increase fertility by restoring pelvic anatomy as well as removing endometrial implants or endometriomas and thereby reducing inflammation [8]. Current data suggest that endometriosis does not negatively impact living birth rates following IVF [22, 23]. For patients over the age of 35 with diminished ovarian reserve, in vitro fertilization (IVF) or intracytoplasmic sperm injection (ICSI) should be offered [24]. In endometriosis patients, a diminished ovarian reserve is particularly evident in women with ovarian endometriosis, in line with the significantly reduced level of anti-Müllerian hormone (AMH). For these patients, fertility preservation through embryo or oocyte cryopreservation may be essential to safeguard their future reproductive potential [25, 26]. Considering that endometriosis subtypes differ in their underlying pathomechanisms, concerns have been raised that a more detailed analysis of their differential impact on infertility and ovarian reserve is required [27].

Endometriosis has been recognized since 2015 as a potential indication for fertility preservation, and pre-surgical counseling is recommended for women of reproductive age who have not yet completed family planning. However, globally recognized guidelines—such as those from ESHRE or ASRM (American Society of Reproductive Medicine)—defining which patients should be offered fertility preservation and which technique should be used are still lacking, and clinical indications therefore remain unclear [28]. The most frequently proposed fertility preservation strategy in patients with endometriosis is oocyte cryopreservation, as it is associated with low morbidity and, once an adequate number of oocytes has been cryopreserved, IVF outcomes are comparable to those of patients undergoing fertility preservation for other indications [29]. Ovarian tissue cryopreservation, a well-established technique in oncology patients, has so far been reported only in a few case series in women with endometriosis and does not currently appear suitable as a standard procedure in this population [30, 31].

Therefore, the aim of the present study was to examine to what extent different forms of endometriosis—despite ovarian endometriosis—influence the ovarian reserve and the number of retrieved oocytes. We were also interested if we could identify patients who might benefit from fertility preservation through oocyte cryopreservation?

## Materials and methods

### Study design

In this retrospective study, we reviewed the medical records from our center for reproductive medicine between January 2014 and February 2025. All patients who underwent follicle punctures as well as laparoscopic surgery to diagnose or rule out endometriosis were included in the present study.A total of 205 patients aged 20–44 years were included in this study.

Endometriosis was confirmed via laparoscopy and supported by histopathological examination of biopsy samples. Laparoscopic surgery was initiated by inserting the primary trocar subumbilically following insufflation of the abdominal cavity with CO_2_ via a Veress needle. Additionally, five-millimeter trocars were placed in the lower abdomen and strategically positioned to allow optimal access to the individually identified endometric lesions. Systemic inspection of the entire abdominal cavity was performed. For staging purposes, the revised American Society of Reproductive Medicine (rASRM) classification system was applied. This scoring system assigns points based on the size and extent of peritoneal and ovarian lesions, as well as the presence and severity of adhesions. The total score can be used to classify endometriosis into one of four stages of severity [32]. To further characterize deep-infiltrating endometriosis (DIE) and adenomyosis uteri, the #ENZIAN classification system was employed [33]. All patients with endometriomas were assigned to the “ovarian endometriosis” group; patients with superficial and deep-infiltrating endometriosis were classified as “deep-infiltrating endometriosis”, while those with only noninvasive peritoneal lesions (< 5 mm lesion depth) were classified as “superficial endometriosis”. Patients who only exhibited adenomyosis were excluded. Patients without histologically confirmed endometriosis form our control group.

The Department of Gynecology and Obstetrics of the University Hospital Würzburg is certified as an endometriosis center level III (clinical and scientific center). Surgical diagnosis of endometriosis was performed by specially trained surgeons. The precise criteria and the distinctions of levels are described in the existing literature [34].

Ethical approval was obtained from the Ethics Committee of Würzburg University (file number: 2025—143).

### Assessing the ovarian reserve and medical history

At the beginning of the reproductive therapy, assessment of the ovarian reserve is routinely performed in our clinic. Between cycle days three and five, a transvaginal ultrasound is conducted to determine the antral follicle count (AFC). The AFC is defined as the total number of ovarian follicles with a visible antral cavity. Follicles measuring between two and ten millimeters in diameter are included in the count. As part of the baseline hormonal assessment, the serum levels of estradiol, follicle-stimulating hormone (FSH), luteinizing hormone (LH), progesterone, prolactin, testosterone, sex hormone-binding globulin (SHGB), and anti-Müllerian hormone (AMH) are measured.

To systematically assess patient history, standardized medical history forms were employed at initial presentation. These forms include detailed inquiries regarding fertility-impairing conditions, the number of previous pregnancies and miscarriages as well as any prior attempts at assisted reproductive therapy.

To evaluate the prognosis of ART success in patients with endometriosis, the EFI was assessed. The EFI score was derived via intraoperative findings as documented in standardized surgical reports, supplemented by relevant clinical data obtained through medical history forms [21].

To evaluate male fertility, semen analysis was performed according to the current World Health Organization (WHO) guidelines. The parameters assessed included sperm count and concentration, progressive and total motility, and sperm morphology. Semen samples are classified as impaired in cases of oligozoospermia, asthenozoospermia or cryptozoospermia [35].

### Controlled ovarian stimulation, follicle puncture, fertilization and embryo quality

Controlled ovarian stimulation was carried out using the protocol described in one of our previous studies [36].

Oocyte retrieval was carried out under either local or general anesthesia. The procedure was performed by a physician specialized in reproductive medicine using transvaginal ultrasound guidance and negative pressure to aspirate the oocytes. In cases of endometriosis, the aspiration system was changed as needed to avoid contact between the follicular aspirate and endometriotic lesions. Mature oocytes were defined as those that reached metaphase II.

The fertilization procedures followed the protocol described in our previous work [36]. ICSI was performed for all couples with impaired male fertility. Fertilization was assessed 16–18 h post-insemination by the presence of two pronuclear-stage oocytes (2PN). The fertilization rate was calculated by dividing the number of 2PN-stage oocytes by the number of mature oocytes. The implantation rate was calculated as the number of detected gestational sacs relative to the number of embryos transferred.

Embryo quality was evaluated using KIDScores™ D3 and D5 for the best fresh embryos, identified to transfer or freeze according to the embryologist’s assessment. KIDScore™ analysis was performed by the Embryoviewer software (Vitrolife), generating KIDScore™ D3 (continuous scale from 1 to 5) or KIDScore™ D5 (continuous scale running from 1 to 9.9)—evaluating morphokinetic markers—according to the day of embryo transfer. Higher scores indicate a greater likelihood of implantation.

### Statistical analysis

Data handling and the statistical analyses were performed using Microsoft Excel 2024 (Microsoft, USA) and SPSS 29 (IBM, USA). For the descriptive statistics, continuous variables are expressed as means ± standard deviations (SD). The categorical variables are expressed as the number of cases (*n*) and percentage of occurrence (%). Chi-square test (*χ*^2^) with Fisher’s exact test was used for categorical variables. Kruskal–Wallis test was used for continuous variables. Subgroup comparisons were corrected by Bonferroni. Statistical significance was defined as a two-sided *p* < 0.05. After rejecting normal distribution using Kolmogorov–Smirnov test, correlation between laboratory values and our clinical data was analyzed using Spearman’s rank correlation, with *p*-values < 0.01 considered statistically significant. Comparisons between high and low AMH groups were performed using Mann–Whitney U test. Statistical significance for these tests was also set at a two-sided *p* < 0.05. Covariates were assessed using ANCOVA and two-sided ANOVA tests, and *p* < 0.05 was considered significant.

## Results

### Sample-description

In this study, we enrolled 118 patients with histologically confirmed endometriosis and 87 without endometriosis (control group, laparoscopically confirmed). The endometriosis group included 36 patients with superficial endometriosis, 40 with deep-infiltrating endometriosis and 42 with ovarian endometriosis. The demographic and baseline characteristics of the study population are displayed in Table 1. Statistically significant differences among the four groups were observed in parity (*p* = 0.045) and the age of the male partner (*p* = 0.024). The control group showed the highest prevalences of tubal factor (*p* = 0.039) and inflammatory bowel disease (*p* = 0.040), as well as the highest testosterone level (*p* = 0.047). The endometriosis subgroups differed significantly in the EFI score (*p* = 0.040), with patients with superficial endometriosis having the highest mean EFI (7.03) and those with ovarian endometriosis having the lowest mean EFI (6.21) (Table 1).

**Table 1:** Demographic and baseline characteristics of the study population. The data are presented as the means (± standard deviations) or *n* (%). The test statistic given was computed by Kruksal-Wallis-Test for nominal rank sums and Chi-Square with Fisher´s exact test for categorial variables. Statistical significance was set at a two-sided *p*-value < 0.05. * *n* = 198, no data available of sperm donors; **arcuate, septate, subseptate, bicornuate, unicornuate, and didelphys uterus

	Superficial endometriosis (*n* = 36)	Deep-infiltrating endometriosis (*n* = 40)	Ovarian endometriosis (*n* = 42)	Control group (*n* = 87)	*p*-value
Baseline characteristics					
Age	34.39 (± 4.82)	32.85 (± 3.30)	34.33 (± 3.70)	34.23 (± 4.00)	0.059
BMI	22.76 (± 3.63)	24.32 (± 4.56)	23.74 (± 3.41)	24.79 (± 4.74)	0.106
Gravida	0.58 (± 1.18)	0.90 (± 1.72)	0.52 (± 0.92)	0.98 (± 1.40)	0.131
Parity	0.08 (± 0.37)	0.35 (± 0.80)	0.19 (± 0.40)	0.36 (± 0.63)	**0.045**
Primary sterility	25 (69.4%)	27 (67.5%)	31 (73.8%)	45 (51.72%)	0.054
Treated before	6 (16.7%)	7 (17.5%)	8 (19.0%)	26 (29.89%)	0.240
Smoker	4 (11.1%)	8 (20.0%)	7 (16.7%)	23 (26.44%)	0.239
EFI	7.03 (± 1.67)	6.48 (± 1.89)	6.21 (± 1.55)		**0.040**
Age of partner of male partner*	37.00 (± 5.68)	35.20 (± 4.38)	36.67 (± 4.57)	38.24 (± 5.62)	**0.024**
Male factor*	11 (30.6%)	18 (45.0%)	14 (33.3%)	33 (37.50%)	0.633
Comorbidities					
Hypothyroidism	9 (25.0%)	13 (32.5%)	12 (28.6%)	27 (31.03%)	0.742
PCO-Syndrome	2 (5.6%)	2 (5.0%)	2 (4.8%)	7 (8.05%)	0.956
Hyperprolactinemia	0 (0.0%)	0 (0.0%)	1 (2.4%)	1 (1.15%)	0.652
Hyperandrogenism	1 (2.8%)	0 (0.0%)	1 (2.4%)	0 (0.00%)	0.345
Insuline Resistance	4 (11.1%)	2 (5.0%)	3 (7.1%)	11 (12.64%)	0.522
STD	0 (0.0%)	1 (2.5%)	2 (4.8%)	0 (0.00%)	0.152
Tubal factor	8 (22.2%)	7 (17.5%)	12 (28.6%)	35 (40.23%)	**0.039**
Uterus anomalies**	4 (11.1%)	10 (25.0%)	6 (14.3%)	11 (12.64%)	0.269
Myoms	7 (19.4%)	4 (10.0%)	5 (11.9%)	12 (13.79%)	0.662
Coagulation disorders	1 (2.8%)	3 (7.5%)	3 (7.1%)	6 (6.90%)	0.813
Pelvic inflammatory disease	3 (8.3%)	3 (7.5%)	3 (7.1%)	8 (9.20%)	0.978
Inflammatory bowel disease	0 (0.0%)	2 (5.0%)	0 (0.0%)	0 (0.00%)	**0.040**
Hormonal assessment					
Progesterone (μg/l)	0.204 (± 0.204)	0.508 (± 1.787)	0.436 (± 0.779)	0.315 (± 0.766)	0.987
FSH (IU/l)	8.23 (± 3.99)	7.40 (± 5.65)	7.80 (± 2.66)	7.61 (± 2.68)	0.207
LH (IU/l)	6.68 (± 3.36)	6.14 (± 3.62)	6.45 (± 3.12)	6.70 (± 4.81)	0.888
PRL (μg/l)	14.31 (± 8.59)	14.16 (± 7.53)	13.99 (± 8.49)	12.92 (± 7.08)	0.805
SHGB (mmol/l)	100.17(± 33.11)	114.38 (± 43.59)	107.00 (± 29.51)	73.41 (± 67.52)	0.689
Testosterone (μg/l)	0.248 (± 0.113)	0.240 (± 0.085)	0.217 (± 0.085)	0.594 (± 2.808)	**0.047**
Estradiol (ng/l)	57.31 (± 55.70)	82.14 (± 84.81)	52.97 (± 42.71)	52.89 (± 31.53)	0.320

We analyzed 61 follicle punctures from patients with superficial endometriosis, 56 from patients with deep-infiltrating endometriosis, 66 follicle punctures from patients with endometriomas and 135 from the control group. 377 embryo transfer procedures, including both fresh and cryopreserved embryos, were analyzed. A total of 55 embryos from patients with superficial endometriosis were evaluated (21 on day 3 and 34 on day 5). Among patients with deep-infiltrating endometriosis, 59 embryos were assessed (14 on day 3 and 45 on day 5). In the ovarian endometriosis group 22 embryos were analyzed on day 3 and 32 on day 5. 34 embryos of the control group were analyzed on day 3 and 133 embryos were analyzed on day 5. Of the 118 patients with endometriosis, 50 had an AMH value lower than 1 ng/ml, whereas 133 had an AMH value higher than 1 ng/ml.

### Correlation between AMH levels and clinical data

**Fig. 1:**
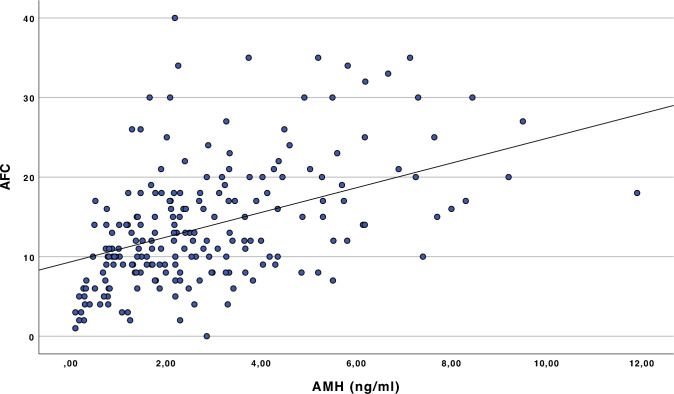
Correlation between AMH (ng/ml) and AFC. The test statistic as given in the figure was computed via Spearman’s rank correlation. The line displays the correlation coefficient

AMH, AFC and the number of retrieved did not show a normal distribution (*p* < 0.001). A statistically significant correlation was observed between AMH levels and the AFC (*r* = 0.505; *p* < 0.001) (Fig. 1). In addition, both AMH levels and the AFC were significantly correlated with the number of retrieved oocytes (*r* = 0.683 and *r* = 0.562; *p* < 0.001 for both comparisons). Furthermore, the number of oocytes correlated significantly with the stimulation dose of hMG [IU] (*r* = − 0.274; *p* < 0.001) and the duration of stimulation (*r* = 0.220; *p* < 0.001), but not with the stimulation dose of FSH [IU] (*r* = 0.028; *p* = 0.610). These correlations support the validity and consistency of the clinical data used in this study.

### Differences in the AMH values and AFCs

**Fig. 2:**
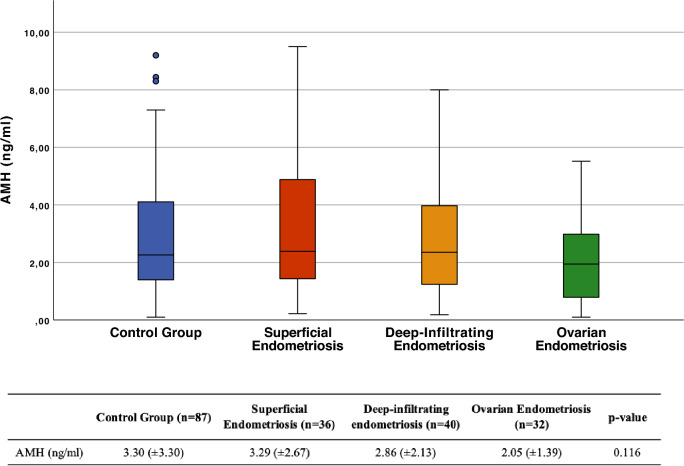
Effect of the endometriosis subtype on AMH-levels The median (prominent line), absolute minimum (lower end of the boxplot), absolute maximum (higher end of the boxplot) are displayed in the figure. The table shows the means ± standard deviation. The test statistic given in the figure was computed via the Kruskal–Wallis test comparing the rank sums. Statistical significance was set at a two-sided *p*-value < 0.05

Figure 2 illustrates the differences in AMH values among the studied groups. A trend toward lower mean AMH levels was observed in patients with ovarian endometriosis (2.05 ng/ml) compared to patients with superficial or deep-infiltrating endometriosis. The control group showed the highest AMH value (3.29 ng/ml). When comparing the different subgroups individually, the largest difference was found between the control group and ovarian endometriosis; however, this difference did not reach statistical significance (*p* = 0.182). Interestingly, the difference between patients with superficial endometriosis and patients without endometriosis was only 0.01 ng/ml. The covariates age (*p* = 0.032), BMI (*p* = 0.015), and a diagnosis of PCOS (*p* < 0.001) had a significant influence on AMH levels, whereas smoking did not (*p* = 0.286). Additionally, a prior cystectomy for endometriomas had no significant impact on AMH-values (*p* = 0.560).

**Fig. 3:**
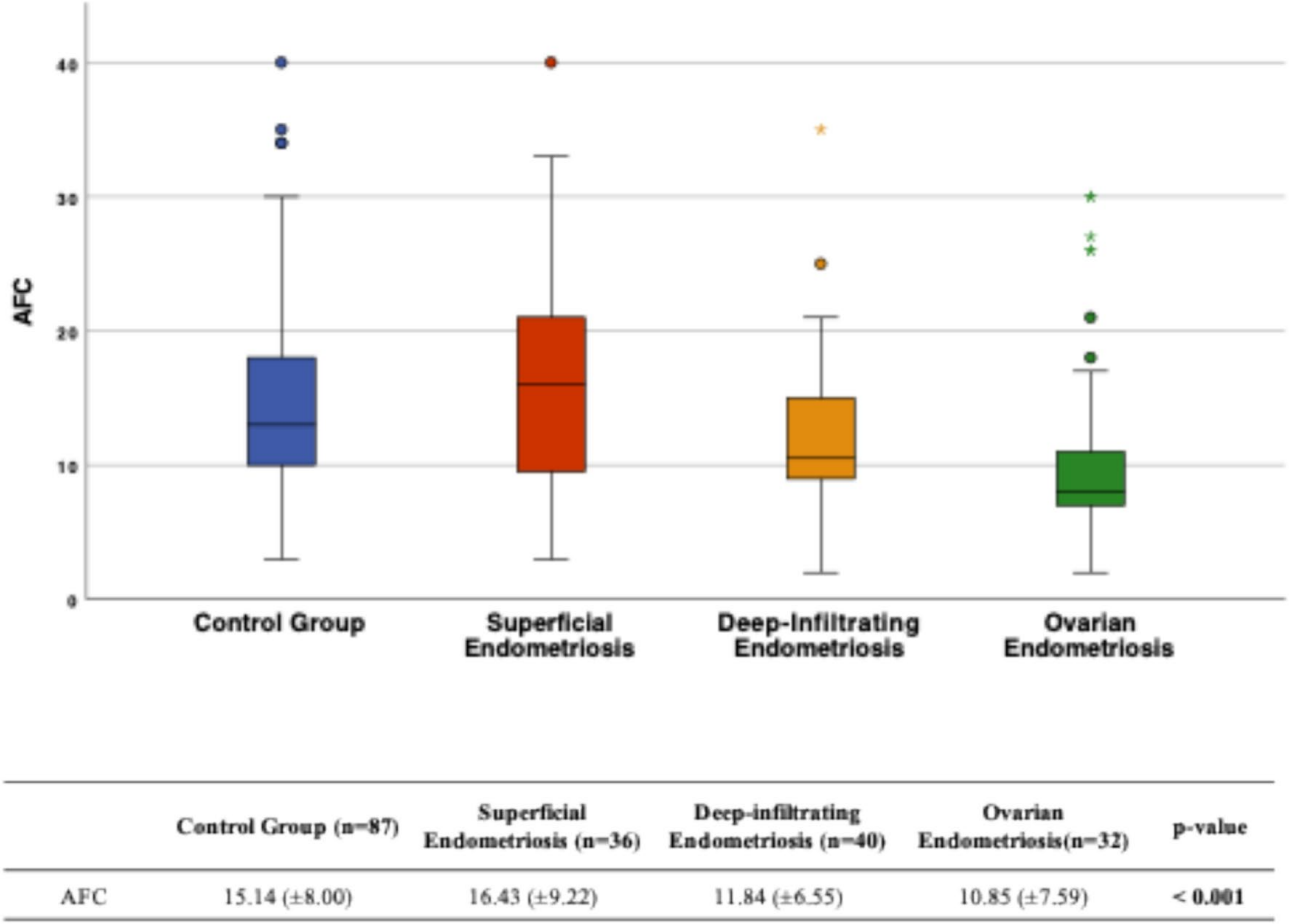
Effect of endometriosis subtypes on AFCs. The median (prominent line), absolute minimum (lower end of the boxplot), absolute maximum (higher end of the boxplot) are displayed in the figure. The table shows the means ± standard deviations. The dots represent the statistical outliers. The test statistic given in the figure was computed via the Kruskal–Wallis test comparing the rank sums. Statistical significance was set at a two-sided *p*-value < 0.05

Figure 3 presents the differences in AFCs between the studied groups. A statistically significant difference was observed between the studied groups (*p* < 0.001). The highest AFC was found in patients with superficial endometriosis (16.43) and the lowest AFC was found in patients with ovarian endometriosis (10.85). Patients with ovarian endometriosis exhibited significantly lower AFCs than patients with superficial endometriosis (*p* = 0.003) and those without endometriosis (*p* = 0.002). The covariates age (*p* = 0.025) and the diagnosis of PCOS (*p* < 0.001) influenced the AFC significantly. The BMI (*p* = 0.233) and smoking status (*p* = 0.770) did not appear to have an impact on the AFC in our study group. Additionally, a prior cystectomy showed no significant impact on the AFC (*p* = 0.426).

### Differences in the number of retrieved oocytes

**Fig. 4:**
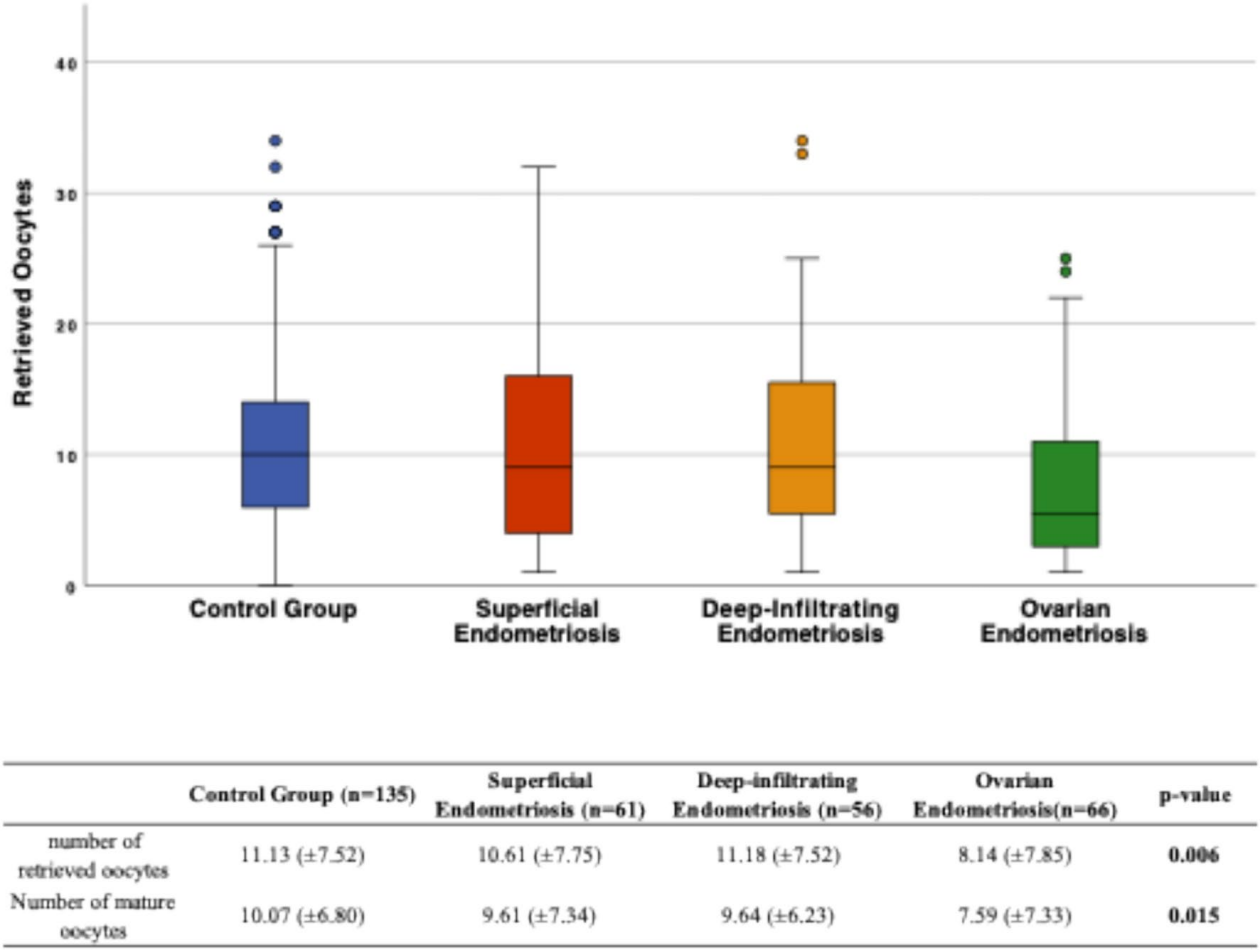
Effect of endometriosis subtype on the number of retrieved oocytes. The median (prominent line), absolute minimum (lower end of the boxplot), and absolute maximum (higher end of the boxplot) for the number of retrieved oocytes are displayed in the figure. The dots represent the statistical outliers. The table shows the means ± standard deviation of the number of retrieved and mature oocytes among the studied groups. The test statistic given in the figure was computed via the Kruskal–Wallis Test comparing the rank sums. Statistical significance was set at a two-sided *p*-value < 0.05

Figure 4 shows the number of retrieved oocytes among the study groups. A statistically significant difference was observed when comparing all groups with each other (*p* = 0.006). The highest number of retrieved oocytes was found in patients with deep-infiltrating endometriosis (11.18), while the lowest number was observed in patients with ovarian endometriosis (8.14). When these two groups were directly compared, the difference remained statistically significant (*p* = 0.032). Another statistically significant difference was observed between patients without endometriosis (11.13) and those with ovarian endometriosis (*p* = 0.005). The number of mature oocytes followed a similar pattern; however, the rate of mature oocytes did not differ significantly between groups. Patients with ovarian endometriosis had the highest maturation rate (0.93) and patients with superficial endometriosis showed the lowest rate (0.90). None of the covariates, mentioned before, showed a significant influence on the numbers of retrieved and mature oocytes as well as on the maturation rate.

When patients with endometriosis were categorized according to AMH level (AMH < 1 ng/ml vs. AMH ≥ 1 ng/ml), a statistically significant difference was found in the number of retrieved oocytes (*p* < 0.001) and the number of mature oocytes (*p* < 0.001); however, AMH had no significant effect on the maturation rate (*p* = 0.293).

### Differences in the fertilization rate

**Fig. 5:**
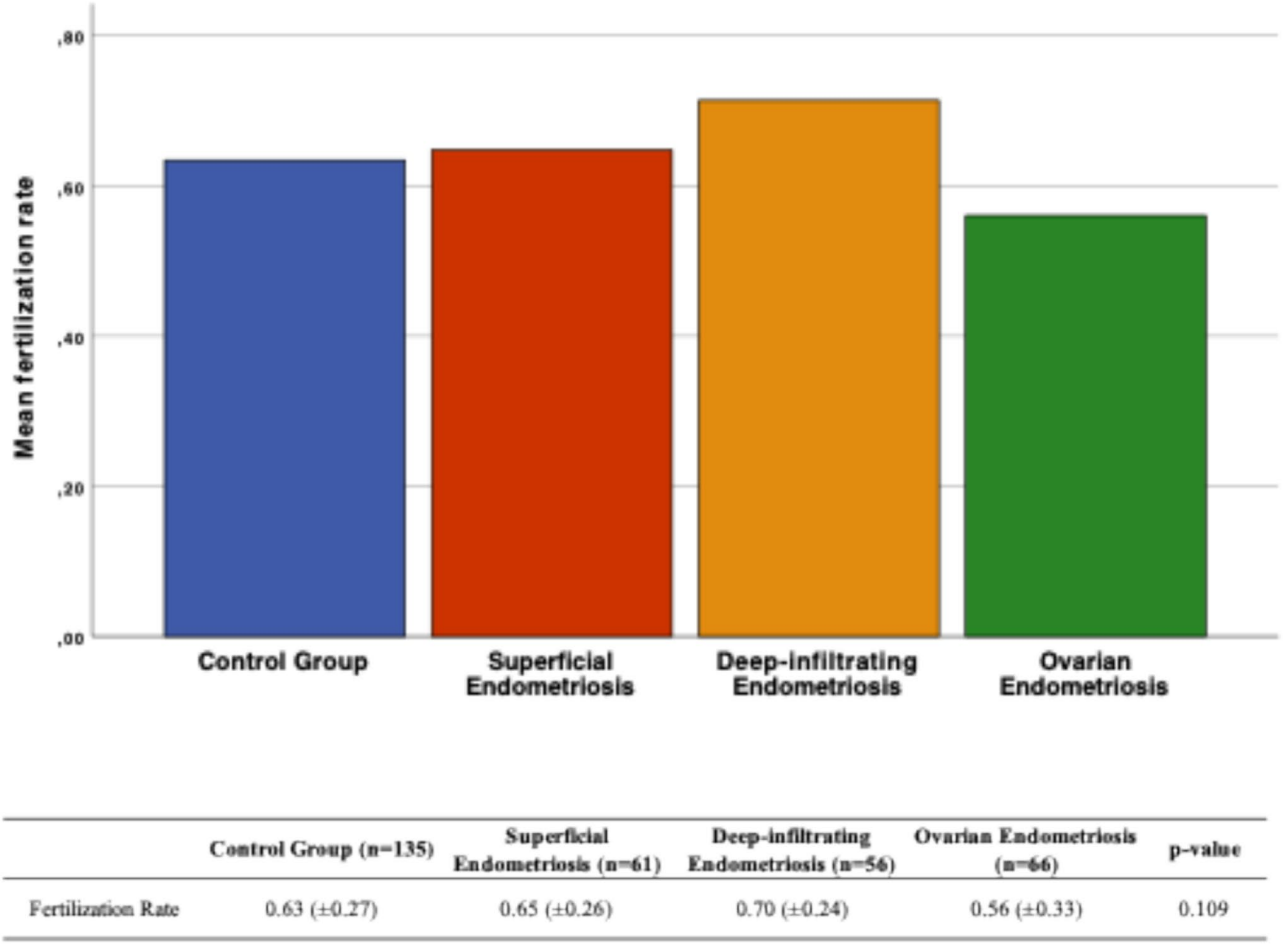
Effect of the endometriosis subtype on the fertilization rate. The bars demonstrate the mean fertilization rate. The table displays the means ± standard deviations among the studied groups. The test statistic given in the figure was computed via the Kruskal–Wallis test comparing the rank sum. Statistical significance was set at a two-sided *p*-value < 0.05

Figure 5 shows the mean fertilization rates across the studied groups. No statistically significant differences were observed between the studied groups (*p* = 0.109). Patients with deep-infiltrating endometriosis had the highest fertilization rate (0.70), whereas those with ovarian endometriosis demonstrated the lowest fertilization rate (0.56). The covariates mentioned before, as well as a male factor, had no impact on the fertilization rate in our study cohort (*p* = 0.303). The implantation rate did not differ significantly between the study groups (*p* = 0.257). The BMI showed a statistically significant association with implantation rate, whereas no significant effects were observed for the other confounding variables.

### Differences in the embryo quality

**Fig. 6:**
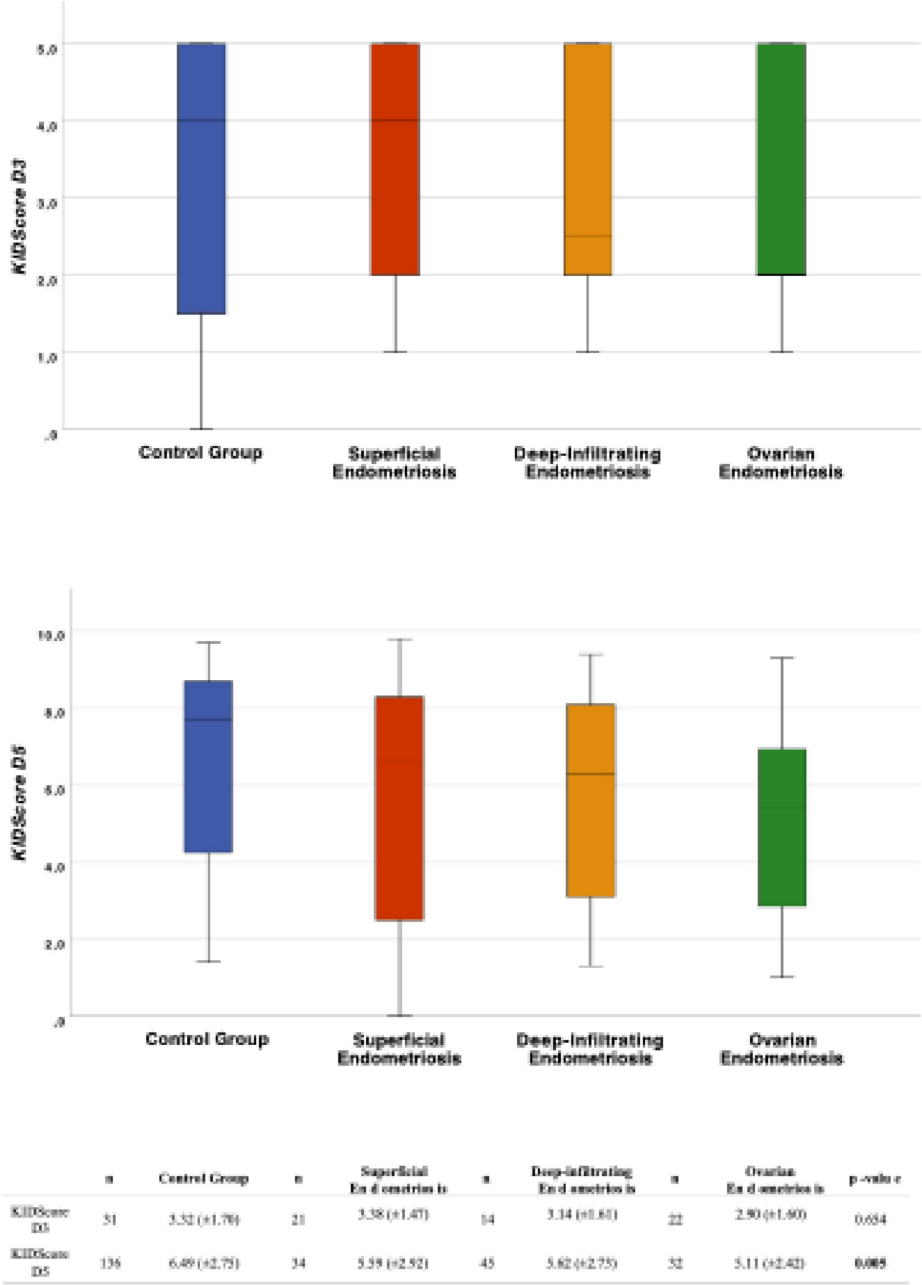
Effects of the endometriosis subtypes on KIDScore™ D3 and D5. The median (prominent line), absolute minimum (lower end of the boxplot), absolute maximum (higher end of the boxplot), mean and number of patients are included in the table. The dots represent the statistical outliers. The table shows the means ± standard deviation of the KIDScore™ D3 and D5 among the studied groups. The test statistic given in the figure was computed via the Kruksal-Wallis Test comparing the rank sum. Statistical significance was set at a two-sided *p*-value < 0.05

Figure 6 illustrates the differences in embryo quality among the anatomical subtypes of endometriosis, assessed using the KIDScore™ on day three or day five. Even though KIDScore™ D3 did not detect a statistically significant difference (*p* = 0.654), KIDScore™ D5 was able to do so (*p* = 0.005). The control group exhibited the highest KIDScore™ D3 (3.69) as well as D5 (6.47). We demonstrated a statistically significant difference in KIDScore™ D5 between patients with ovarian endometriosis (5.11) and those without endometriosis (*p* = 0.010). The covariates that were mentioned early did not affect the KIDScores™ D3 and D5.

## Discussion

In this study, we analyzed the impact of different endometriosis subtypes on ovarian reserve and IVF/ ICSI outcomes to determine which patients might benefit the most from medical freezing. We demonstrated that patients with ovarian endometriosis exhibit a significantly diminished ovarian reserve, as reflected by lower AMH levels, a reduced AFC, and fewer retrieved and mature oocytes. They not only exhibit a diminished ovarian reserve but also exhibit statistically significant lower embryo quality on day 5. In contrast, the proportion of mature oocytes and the fertilization rate did not differ between the study groups.

Our study addresses an important gap in the literature, as most previous studies compared only endometriosis patients to controls without distinguishing between subtypes. We underscore that subtype differentiation is essential for accurate assessment and personalization of fertility preservation planning.

The need to preserve fertility—particularly for patients with endometriomas—is reinforced by our findings. These patients presented with lower AMH levels and significantly lower AFCs, both of which are well-established predictors of ovarian reserve and ovarian response in stimulated cycles [37, 38]. Interestingly, although AMH, the AFC, and the number of retrieved oocytes were strongly correlated, the AMH values alone did not differ significantly between the groups, whereas AFC and the number of retrieved and mature oocytes did. An explanation for that could be the small sample size. Our analysis of patients with low versus high AMH further confirmed that AMH and the AFC serve as quantitative indicators: while the number of mature oocytes differed between groups, the rate of mature oocytes did not.

Our results are consistent with previous findings from Kasapoglu et al. [39] and Muzii et al. [26], who also reported reduced AMH levels in women with endometriomas. This decline in ovarian reserve may be attributed to local pathological changes, including fibrosis, the loss of cortical stroma, and cytotoxic oxidative stress caused by high iron concentration resulting from hemolysis [13, 17, 18]. Ferrero et al. further support this hypothesis, showing significantly lower AFCs and a lower number of retrieved oocytes from ovaries with large endometriomas than from healthy contralateral ovaries. Interestingly, we did not observe a lower fertilization rate in these patients, suggesting that oocyte quality may be preserved, and that quantity is the primary limiting factor. AMH and the AFC did not correlate with fertilization rate, as deep-infiltrating endometriosis had lower AMH and AFC values than those with superficial endometriosis but had a higher fertilization rate. This indicates that fertilization potential may be influenced by factors beyond the ovarian reserve.

All patients with both ovarian and deep-infiltrating endometriosis were classified as ovarian endometriosis in our study, but only a trend toward a diminished ovarian reserve was observed among patients with isolated deep-infiltrating endometriosis. One prior study also distinguished between patients with endometrioma and those with deep-infiltrating endometriosis and demonstrated that patients with deep-infiltrating endometriosis had significantly lower AMH-values than patients exhibiting both subtypes [40]. The absence of statistical significance in our study is most likely due to limited statistical power resulting from the small sample size.

In contrast, patients with superficial endometriosis—who represent most cases of mild endometriosis—did not exhibit signs of diminished ovarian reserve. Our results align with those of Lessans et al. [41], who also reported preserved AMH and AFC in this population. Additionally, we observed no significant differences in the number of retrieved oocytes or fertilization rates among these patients. Xu et al. proposed that impaired fertility in these patients may result from qualitative, rather than quantitative factors which were identified by electron microscopy [42]. Additionally, altered peritoneal fluid composition, which affects EGF and IGF-I, compromises embryo development through contact with the uterine cavity [16]. However, based on our data—which revealed no significant difference in embryo quality across the anatomical subtypes—this effect likely influences all forms of endometriosis rather than being specific to superficial endometriosis. In the clinical evaluation of whether medical cryopreservation is an appropriate option for patients, these factors must be considered, as oocyte quality declines not only as a result of endometriosis but also with increasing age [43].

In patients with deep-infiltrating endometriosis, the AMH values and AFCs were lower than those in patients with superficial endometriosis, but the number of retrieved and mature oocytes was not significantly reduced, indicating a preserved ovarian function despite anatomical disruption. Previous studies, such as that by Papaleo et al. [44], reported a reduced oocyte number in DIE patients, but their cohorts also included patients with endometriomas. Adhesions and mechanical interference with oocyte release and tubal pick-up likely contribute to infertility in DIE patients, suggesting that IVF/ICSI may overcome these barriers.

To our knowledge, no study has compared the impact of different anatomical endometriosis subtypes on embryo quality using the KIDScore™ D3 or D5 algorithm, which uses morphokinetic annotations. Showing that embryos from patients with ovarian endometriosis exhibit lower KIDScores™ on day 5, as a marker of their quality, we suggest that not only quantitative but also qualitative reproductive parameters may be compromised. This aligns with the finding of Ding et al., who reported a higher apoptosis rate in mouse embryos exposed to peritoneal fluid from endometriosis patients [16].

Many authors have validated the mechanisms through which endometriosis impairs fertility, highlighting both qualitative and quantitative factors. However, few studies have differentiated by subtype. Our study focused primarily on ovarian reserve as a quantitative marker and demonstrated the clinical importance of considering subtype in fertility preservation planning. However, we were also able to demonstrate differences in the embryo quality between different types of endometriosis as ovarian endometriosis seems the only subtype with a significant decrease. These patients also experience a significant decline in ovarian reserve and would benefit most from early fertility preservation to reach comparable IVF results [29]. In contrast, patients with superficial and deep-infiltrating endometriosis do not exhibit the same quantitative decline, suggesting that qualitative factors, mechanical barriers or local inflammation may play a more substantial role in these subtypes in spontaneous conception. Our results highlight the heterogeneity of endometriosis and emphasize the importance of considering different prognostic factors when identifying the most appropriate treatment approach for each patient. In the future, these data should be incorporated into clinical guidelines, and more accurate tools capable of capturing the complexity of endometriosis need to be developed to better predict the potential benefit of fertility preservation.

A major strength of our study is the well-defined population: all patients underwent laparoscopy, allowing precise confirmation of endometriosis and its subtype. The study groups were well-matched, and even though no covariate analyses were performed the influence of other infertility-related comorbidities was balanced among the study groups. Both AMH and the AFC—robust markers of ovarian reserve—were used [37, 38]. The subjective nature of AFC measurement is mitigated by its strong correlation with AMH values and the number of retrieved oocytes, supporting the internal validity of our data.

However, this study has limitations. The retrospective design inherently limits causal interpretation [45]. As the data already exist, the impact of missing data is low. Patient histories were collected via self-reports, introducing potential reporting bias.

It is important to emphasize that our cohort represents women with established infertility undergoing ART, and thus does not reflect the population in whom fertility preservation is typically performed. Consequently, ART outcomes should not be interpreted as a proxy for fertility preservation efficacy. Instead, our results provide indirect evidence of compromised reproductive potential, as reflected by reduced oocyte yield and embryo quality. While these findings may suggest that earlier intervention could be beneficial, this requires confirmation in prospective studies.

Interestingly, we did not observe a significant impact of prior cystectomy on AMH levels or AFC when evaluating covariates influencing these parameters. This finding contrasts with existing literature, which suggests that cystectomy may have a greater detrimental effect on ovarian reserve than the presence of endometriomas itself [14]. Therefore, these results should be interpreted with caution. A possible explanation is the relatively small sample size, as well as the fact that all surgeries were performed in a tertiary endometriosis center by specialized surgeons, potentially minimizing surgical damage to ovarian tissue. Accordingly, it remains difficult to distinguish whether diminished ovarian reserve is attributable to the disease itself or to iatrogenic factors. Further studies are necessary which differentiate between operated and not operated endometriomas in comparison to other endometriosis subtypes and evaluate if a Medical Freezing prior to a cystectomy could be beneficial. We did not account for prior endocrine therapy prior to reproductive treatment, which may represent a confounding factor given its association with improved outcomes [46]. A very conservative and careful surgical approach, as well as endocrine therapy, may represent potential alternatives to cryopreservation for fertility preservation. However, it should be noted that a recent meta-analysis reported a recurrence rate of endometriomas of approximately 27% within 24 months in the absence of hormonal therapy [47]. In contrast, recurrence rates decrease to 6–14% with hormonal therapy [48]. Nevertheless, observational data indicate that more than half of patients report a negative attitude toward endocrine therapy [49]. Furthermore, the effectiveness of progestin-based therapy depends on progesterone receptor expression, which is frequently reduced or dysregulated in endometriotic lesions [50]. Considering these factors, along with current ESHRE guidelines that discourage surgical intervention for ovarian endometriomas, we suggest that elective oocyte cryopreservation (“Medical Freezing”) may represent a safer and more acceptable option for fertility preservation in this patient population. Especially, age and the diagnosis of PCOS, as covariates, had a significant influence on AMH levels and AFC but these findings are in line with the existing literature [51, 52]. We did not account whether IVF or ICSI was used for fertilization, as male factor was equally distributed between the study groups, and stratification would have resulted in insufficient case numbers. The size of the endometrioma was also not assessed, although it may influence ovarian reserve. This was a single-center study with a limited sample size resulting in small subgroups, which may restrict generalizability. Future prospective, multicenter studies with larger cohorts are needed to validate our findings.

## Conclusion

Our study emphasizes that accurate assessment of endometriosis subtypes is essential, as patients with different subtypes exhibit distinct ovarian reserve and embryo quality at the time of surgery. To address these differences, we propose the development of subtype-specific fertility preservation programs. For patients with ovarian endometriosis, characterized by diminished ovarian reserve and reduced embryo quality, early medical freezing may help preserve a higher number of oocytes of good quality. In contrast, for patients with superficial or deep-infiltrating endometriosis, additional factors should be considered when considering the suitable approach for fertility preservation. Overall, a tailored, individualized approach is necessary to optimize reproductive outcomes in this patient population.

## Data Availability

The datasets generated and/or analyzed during the current study are available from the corresponding author upon reasonable request.
